# Potential of Rice-Flour Jelly Made from High-Amylose Rice as a Dysphagia Diet: Evaluation of Pharyngeal Residue by FEES

**DOI:** 10.1007/s00455-022-10529-y

**Published:** 2022-10-15

**Authors:** Misao Tsubokawa, Junko Fujitani, Kanae Ashida, Mika Hayase, Namiko Kobayashi, Chika Horita, Masafumi Sakashita, Takahiro Tokunaga, Tadanori Hamano, Ken-ichiro Kikuta, Shigeharu Fujieda

**Affiliations:** 1 Department of Rehabilitation Medicine, Fukui University Hospital, 23-3, Matsuoka Shimoaizuki, Eiheiji-cho, Yoshida-gun, Fukui 910-1193 Japan; 2 Department of Rehabilitation, National Center for Global Health and Medicine, 1-21-1 Toyama, Shinjuku-ku, Tokyo, 162-8655 Japan; 3 Institute of Food Research, National Agriculture and Food Research Organization, 2-1-12, Kannondai, Tsukuba City, Ibaraki 305-8642 Japan; 4 Nutrition Department, Fukui University Hospital, 23-3, Matsuoka Shimoaizuki, Eiheiji-cho, Yoshida-gun, Fukui, 910-1193 Japan; 5 Department of Otorhinolaryngology and Head & Neck Surgery, University of Fukui, 23-3, Matsuoka Shimoaizuki, Eiheiji-cho, Yoshida-gun, Fukui, 910-1193 Japan; 6 Research Promotion Office, Shinseikai Toyama Hospital, 89-10, Shimowaka, Imizu City, Toyama 939-0243 Japan; 7 Medical Research Support Center, Fukui University Hospital, 23-3, Matsuoka Shimoaizuki, Eiheiji-cho, Yoshida-gun, Fukui, 910-1193 Japan; 8 Department of Neurology, University of Fukui, 23-3, Matsuoka Shimoaizuki, Eiheiji-cho, Yoshida-gun, Fukui, 910-1193 Japan; 9 Department of Neurosurgery, University of Fukui, 23-3, Matsuoka Shimoaizuki, Eiheiji-cho, Yoshida-gun, Fukui, 910-1193 Japan

**Keywords:** Dysphagia diet, Fiberoptic endoscopic evaluation of swallowing, Pharyngeal residue, Rice-flour jelly

## Abstract

Dysphagia diets are recommended to prevent choking and aspiration in people with dysphagia; however, rice-porridge and mashed rice-porridge, which are used as staple foods for people with dysphagia in Japan, are time-consuming to prepare. The National Agriculture and Food Research Organization has found jelly-like food products made from high-amylose rice-flour (rice-flour jelly) to be easy to prepare with a texture suitable for dysphagia diets. To investigate the potential of rice-flour jelly for the dysphagia diet, we evaluated the amount of pharyngeal residue after swallowing rice-flour jelly using fiberoptic endoscopic evaluation of swallowing and compared it with those of rice-porridge, mashed rice-porridge, and fruit jelly. We enrolled 70 participants (43 males and 27 females, aged 32–96 years, median 74.5 years) and evaluated their pharyngeal residue using the Yale Pharyngeal Residue Severity Rating Scale which includes five levels from I (none) to V (severe). Statistical analysis showed that level I was more common in fruit jelly for vallecula residue and pyriform sinus residue, and level III (mild) was more common in rice-porridge for vallecula residue (*p* < 0.05). No differences of pharyngeal residue were found in rice-flour jelly or mashed rice-porridge. No significant difference was observed in the number of participants with laryngeal penetration or aspiration. Therefore, rice-flour jelly is a suitable alternative to rice-porridge as a staple food for people with dysphagia in terms of food texture.

## Introduction

Approximately 8% of the worldwide population has dysphagia [1]. In Japan, the percentage of the population > 65 years of age was as high as 28.4% in 2018, and aspiration pneumonia was responsible for 3% of deaths [2]. Thus, dysphagia diets are recommended to prevent choking and aspiration in people with dysphagia [3, 4], and the need for dysphagia diets is expected to increase as the number of people with dysphagia increases.

Rice is the staple food of the Japanese people. It is equivalent to the International Dysphagia Diet Standardisation Initiative (IDDSI) 6, which describes soft and bite-sized foods that must be chewed before swallowing [4]. Therefore, to use rice in the dysphagia diet for easy swallowing, rice-porridge or mashed rice-porridge must be prepared according to the IDDSI 5 (minced & moist foods) or IDDSI 4 (pureed foods), respectively [4]. Rice is cooked by boiling with water, and rice-porridge is cooked with five to 10 times the amount of water used for cooking rice. Moreover, mashed rice-porridge requires additional mashing of the cooked rice-porridge, which hinders the preparation of rice-based foods for the dysphagia diet at home or in facilities. Thus, an easy-to-prepare, rice-based dysphagia diet is desired to enable the safe oral intake of rice for those with dysphagia.

The National Agriculture and Food Research Organization (NARO) has discovered a jelly-like diet with low adhesion that can be easily prepared by adding 10 times the amount of water to high-amylose rice-flour with an amylose content of ≥ 25% and boiling it [5]. If this jelly-like food made from rice-flour jelly is suitable for those with dysphagia, it would enable the easy preparation of a rice-based dysphagia diet. In this study, we aimed to investigate the potential of rice-flour jelly for the dysphagia diet by observing the swallowing process of the jelly in those with dysphagia using fiberoptic endoscopic evaluation of swallowing (FEES). Additionally, the amount of pharyngeal residue after swallowing rice-flour jelly was compared with that of rice-porridge, mashed rice-porridge, and fruit jelly, which are currently used for the dysphagia diet in Japan [6].

## Methods

### Participants

A total of 272 patients underwent FEES for the evaluation of swallowing functions at our hospital between April 1 and December 20, 2021. Of these, 70 patients who provided consent in writing and had data available for evaluation regarding the study diets (rice-flour jelly, rice-porridge, mashed rice-porridge, and fruit jelly) were included in the study.

### FEES Procedure

FEES was performed using an Olympus ENF-V3 and Olympus OTV-S12 (Tokyo, Japan) according to the manual of the Japanese Society for Dysphagia Rehabilitation [7]. The condition of the pharyngeal and laryngeal regions was observed after insertion of the endoscope, followed by examinations of the swallowing ability of the test foods (colored thickened water, mashed rice-porridge, fruit jelly, rice-porridge, rice-flour jelly, chopped diet, rice, and colored water, in that order). The selection of test foods and the order of examination was altered according to the state of swallowing. A volume of 1–5 cm^3^ of colored thickened water and colored water using a syringe and one teaspoon (about 3 g) of the other test foods was examined. When pharyngeal residue was observed, multiple swallows, alternate swallows, or head rotation was performed to remove the residue before evaluation of the next test food. When aspiration or laryngeal penetration was observed, throat clearing was encouraged by coughing. When aspiration, laryngeal penetration, or pharyngeal residue was not self-cleared, suction was performed, and the examination was finished. The entire process was recorded on a hard disk drive using HDMI/analog capture (I･O DATA, Kanazawa, Japan).

### Study Diets

Rice-flour jelly, rice-porridge, mashed rice-porridge, and fruit jelly were used as the study diets. The rice-flour jelly, rice-porridge, and mashed rice-porridge were prepared at the nutrition department of Fukui University Hospital. The rice-porridge and mashed rice-porridge were the same as those provided to inpatients requiring dysphagia diets at Fukui University Hospital. Rice-flour jelly was prepared by mixing 30 g of high-amylose rice-flour with 40 mL water, adding another 275 mL of boiling water, stirring for approximately 30 s, heating in a microwave oven (Toshiba, Tokyo, Japan) at 500 W for 2 min, and then cooling at 4℃ for 2 h [5]. Rice-porridge was prepared by boiling 1 kg of rice and 9 L of water for 70 min using an electric three-dimensional rice cooker CRAE-150 (Comet Kato, Inazawa, Japan) and removing the supernatant liquid after boiling. Mashed rice-porridge was prepared by mashing rice-porridge for 2 min with an MX-153G professional mixer (Panasonic, Tokyo, Japan). For the fruit jelly, we used Procca Zn Grape (Nutri, Yokkaichi, Japan), which has been approved by the Japan Consumer Affairs Agency as a food for people with dysphagia.

### Outcome Measures

The functional oral intake scale (FOIS) was used to rate the subjects’ diet levels [8] (Appendix 1). The pre-test FOIS was the diet level before the FEES examination, which was extracted from the medical records. The post-test FOIS was determined based on the results of the FEES examination by the physician in charge, rehabilitation physician, and speech-language pathologist [8–10].

### Texture of Study Foods

The food texture (firmness, cohesiveness, and adhesiveness) of the rice-flour jelly, rice-porridge, and mashed rice-porridge was measured by NARO using a RHEONERII RE2-33005C creep meter (Yamaden, Tokyo, Japan) in accordance with the test method for diets for people with dysphagia by the Consumer Affairs Agency [11]. The sample was filled into a stainless steel Petri dish 40 mm in diameter and 15 mm high, fixed to a sample stand, and compressed twice at a compression speed of 10 mm/s and a clearance of 5 mm using a plastic plunger 20 mm in diameter and 8 mm high at 24 °C. Because the mashed rice-porridge was in a liquid state with high fluidity, it was not sufficient to evaluate the texture only by measuring firmness, adhesion, and cohesion. Therefore, for the mashed rice-porridge, the viscosity was measured using a DVNext cone-plate rotational viscometer (AMETEK Brookfield Inc., Middleboro, USA). The viscometer commenced rotating at a speed of 50 s^−1^ at 20 °C, and the viscosity was measured 2 min later [11]. Each food texture metric was measured three times, and the mean and standard deviation values were presented. The texture of the fruit jelly was taken from the literature [12].

### Evaluation of Pharyngeal Residue

First, we extracted and edited the images of swallowing study diets from the recorded FESS images of participants. Using edited images, three raters (one physician with > 15 years of experience and two speech-language pathologists with > 10 years of experience) evaluated the residue volume of the vallecula and pyriform sinuses based on the Yale Pharyngeal Residue Severity Rating Scale (YPRSRS) on five levels: I (none), II (trace), III (mild), IV (moderate), and V (severe) [13, 14] (Appendix 2). When the three raters agreed upon the level, that level was selected; when the three evaluated levels were divided two to one, the level chosen by the majority was selected; and when the three evaluated levels were different, the middle level was selected.

### Statistical Analysis

The vallecula and pyriform sinuses residue of the study diets were compared using the χ^2^ test and residual analysis. The inter-rater reliability was assessed using the kappa statistics. IBM SPSS Statistics version 22 (IBM, Chicago, USA) was used for statistical analysis with a significance level of *p* < 0.05.

### Ethical Considerations

The University of Fukui Ethics Review Committee approved this study (Approval No. 20200179) and participants provided written consent after the study protocol was explained both in writing and orally.

## Results

### Participant Characteristics

Table 1 shows a summary of the participant characteristics. All participants were inpatients. Participants with neuromuscular diseases were admitted for detailed examination, while the rest were admitted for treatment. Participants with acute brain diseases were the most common, including 28 with cerebrovascular diseases, two with postoperative brain tumors, and two with encephalitis. Furthermore, nine participants had head and neck cancer, including five undergoing chemoradiotherapy and four in a postoperative state. Moreover, 17 participants had other diseases including seven with cardiac disease, four with pneumonia, and six in a post-fracture state.

**Table 1:** Participant characteristics

	Total (*n* = 70)	Diagnosis			
		Acute brain disease (*n* = 32)	Neuromuscular disease (*n* = 12)	Head and neck cancer (*n* = 9)	Others (*n* = 17)
Age (years), median (range)	74.5 (32–96)	78.5 (37–92)	68 (32–84)	71 (59–96)	81 (53–92)
Male/female (*n*)	43/27	14/18	9/3	6/3	14/3
Tube feeding (*n*)	16	6	2	3	5
Tracheotomy (*n*)	3	0	1	1	1

### Diet Level

**Fig. 1:**
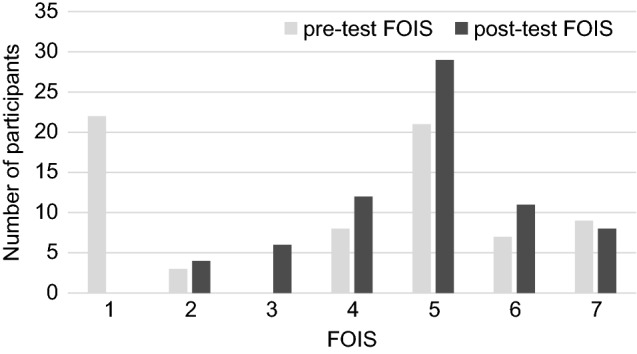
Functional oral intake scale (FOIS) of participants. Pre-test FOIS is the diet level before FEES examination. Post-test FOIS is the diet level based on the results of the FEES examination

The pre- and post-test FOIS for all participants are shown in Fig. 1. The pre-test FOIS results showed 54 participants with a scale of ≤ 5, and the post-test FOIS results showed 51 participants with a scale of ≤ 5.

### Texture of the Study Foods

The textures of the study foods are shown in Table 2. The viscosity of mashed rice-porridge was 509 ± 44 mPa s. Because the fruit jelly was a commercial product, the metrics of its texture were taken from the report by Kayashita et al. [12].

**Table 2:** Food texture of rice-flour jelly, rice-porridge, and mashed rice-porridge measured three times using a creep meter

	Temperature (°C)	Firmness (N/m^2^) mean ± SD	Cohesiveness mean ± SD	Adhesiveness (J/m^3^) mean ± SD
Rice-flour jelly	24	4896 ± 739	0.62 ± 0.04	758 ± 95
Rice-porridge	24	5188 ± 533	0.62 ± 0.09	1296 ± 236
Mashed rice-porridge	24	151 ± 5	0.78 ± 0.03	72 ± 4
Fruit jelly	20	3918	0.27	45

The mean and standard deviation (SD) are presented. The properties of fruit jelly were extracted from Ref. [12]

### Residue of Study Foods in the Vallecula and Pyriform Sinuses

The pharyngeal residues of the foods after swallowing were evaluated by the YPRSRS [13, 14] (Table 3). The statistical analysis showed that the p-values for the vallecula and pyriform sinus residue were both < 0.001, suggesting that there is a difference in the amount of residue among the study diets for both the vallecula and pyriform sinuses (*p* < 0.05). The results of residual analysis showed that level I was more common in fruit jelly for vallecula and pyriform sinus residue, and level III was more common in rice-porridge for vallecula residue (*p* < 0.05). No differences in the pharyngeal residue were found for mashed rice-porridge or rice-flour jelly. After excluding participants with a high risk of aspiration, level V was not observed. Inter-rater reliability for the three raters on all assessments was moderate, *κ* = 0.5002. Laryngeal penetration was observed in five participants with rice-flour jelly, seven with rice-porridge, 14 with mashed rice-porridge, and four with fruit jelly. Aspiration was observed in two participants with rice-porridge and one with fruit jelly. No participants developed pneumonia throughout the study period.

**Table 3:** Distribution of Yale Pharyngeal Residue Severity Rating Scale of study diets

	Yale Pharyngeal Residue Severity Rating Scale				
	I	II	III	IV	V
Residue of vallecula:					
Rice-flour jelly	33 (47.1%)	27 (38.6%)	8 (11.4%)	2 (2.9%)	0 (0.0%)
Rice-porridge	21 (30.0%)	28 (40.0%)	18 (25.7%)^*^	3 (4.3%)	0 (0.0%)
Mashed rice-porridge	27 (38.6%)	31 (44.3%)	11 (15.7%)	1 (1.4%)	0 (0.0%)
Fruit jelly	54 (77.1%)^*^	14 (20.0%)	2 (2.9%)	0 (0.0%)	0 (0.0%)
Residue of pyriform sinuses:					
Rice-flour jelly	44 (62.9%)	25 (35.7%)	1 (1.4%)	0 (0.0%)	0 (0.0%)
Rice-porridge	40 (57.1%)	22 (31.4%)	7 (10.0%)	1 (1.4%)	0 (0.0%)
Mashed rice-porridge	42 (60.0%)	18 (25.7%)	7 (10.0%)	3 (4.3%)	0 (0.0%)
Fruit jelly	63 (90.0%)^*^	4 (5.7%)	3 (4.3%)	0 (0.0%)	0 (0.0%)

^*^Statistical analysis demonstrated that level I in jelly for vallecula and pyriform sinuses and level III in rice-porridge for vallecula are more common (*p* < 0.05)

## Discussion

For people with dysphagia to continue oral intake safely and efficiently, their swallowing function must be assessed, and they must be provided a dysphagia diet and feeding methods appropriate to their function [1, 3]. NARO has developed rice-flour jelly, which can be easily prepared, and the purpose of this study was to investigate the suitability of this rice-flour jelly for the dysphagia diet. We observed that the pharyngeal residue of rice-flour jelly did not differ from that of mashed rice-porridge and was less than that of rice-porridge. Moreover, laryngeal penetration or aspiration of rice-flour jelly was less common than that of rice-porridge and mashed rice-porridge. Therefore, we have determined that rice-flour jelly could be used as an alternative to rice-porridge in the dysphagia diet.

To assess the feeding and swallowing functions of people with dysphagia, the water or diet tests are usually used [15], but for a detailed evaluation, a videofluoroscopic swallowing study (VFSS) and FEES are useful. VFSS and FEES can visualize the presence of laryngeal penetration, aspiration, and pharyngeal residue after swallowing [16, 17], which allows a choice of the appropriate dysphagia diet and feeding method for individual patients. While avoiding diets that will be penetrated or aspirated is vital when considering the appropriate diet for those with dysphagia, it is also necessary to avoid diets with substantial pharyngeal residue. Previous studies have suggested that pharyngeal residue reduces the efficiency of diet intake [18] and increases the risk of laryngeal penetration and aspiration [19, 20]. Therefore, we examined the pharyngeal residue of rice-flour jelly and compared it with that of rice-porridge or mashed rice-porridge using FEES. FEES allows testing without the use of contrast media, which affects the texture of the foods.

In our study, three raters individually reviewed recorded images and evaluated the level of pharyngeal residue according to the YPRSPS, which has been used in many studies [13, 14]. In 43–78% of cases, all three raters agreed on the evaluation, and in 93–100% of cases two of the three raters agreed, and inter-rater reliability was moderate, *κ* = 0.5002, which is a low agreement rate compared to previous reports [9, 10]. This could be because many of the participants had unstable medical conditions, such as acute brain disease, that required quick evaluation, sometimes resulting in unclear images.

While many foods have been used in dysphagia diets recently, few studies have reported the swallowing status of people with dysphagia eating these foods [3]. Our study has provided insight into the pharyngeal residue from dysphagia diets. Comparing the texture of rice-porridge and rice-flour jelly, the firmness and cohesiveness were similar, but the adhesiveness of rice-porridge was higher. The greater adhesiveness may have caused the higher vallecula residues from the rice-porridge. For mashed rice-porridge and rice-flour jelly, the number of participants in each YPRSRS level was nearly the same, while the YPRSRS level for mashed rice-porridge and that for rice-flour jelly often differed among the same participants. In some, the YPRSRS level for mashed rice-porridge was higher than for rice-flour jelly, while the opposite was true for others. Thus, the residual portion and amount of food depend not only on the pathological condition of dysphagia, but also food texture. By increasing the number of cases under investigation and formulating clearer criteria for participation, the relationship between pharyngeal residue and food texture may be elucidated in more detail. In the future, we would like to clarify the criteria for choosing mashed rice-porridge or rice-flour jelly.

In our study, the amount of pharyngeal residue of fruit jelly, which is widely used in Japan as a training diet for people with severe dysphagia [11, 12], was less than that of the other study diets. Thus, we reaffirmed that this fruit jelly is easy to swallow, but the fruit jelly is difficult to use as a staple food in Japan.

The rice-flour jelly is easier to prepare than rice-porridge or mashed rice-porridge and using rice-flour jelly in the dysphagia diet will help reduce the burden and labor of caregivers. There are many countries, not only Japan, where rice is a staple food [21]. When those countries also have aging populations, rice-based dysphagia diets might be needed. Additionally, rice-flour jelly is gluten-free and can be used for those with wheat allergies [22]. Therefore, we believe that rice-flour jelly will be widely useful as a rice-based dysphagia diet. Furthermore, the high-amylose rice used in this rice-flour jelly has been reported to have a lower glycemic index, resulting in a lower increase in postprandial blood glucose [22]. In the future, we expect that high-amylose rice-flour jelly could be used as a therapeutic, dysphagia diet.

## Study Limitations

For safety reasons, most of the participants in our study had only mild dysphagia. Thus, future studies should confirm whether people with moderate dysphagia can use rice-flour jelly as part of their dysphagia diet.

## Conclusion

Because rice-porridge leaves more residue in the vallecula than rice-flour jelly, rice-flour jelly could be used as an alternative to rice-porridge as a staple food for people with dysphagia in terms of food texture.

## Appendix 1

See Table 4.

**Table 4:** Functional oral intake scale (FOIS)

Level 1	Nothing by mouth
Level 2	Tube dependent with minimal attempts of food or liquid
Level 3	Tube dependent with consistent oral intake of food or liquid
Level 4	Total oral diet of a single consistency
Level 5	Total oral diet with multiple consistencies, but requiring special preparation or compensations
Level 6	Total oral diet with multiple consistencies without special preparation, but with specific food limitations
Level 7	Total oral diet with no restrictions

## Appendix 2

See Table 5.

**Table 5:** Yale Pharyngeal Residue Severity Rating Scale

Vallecula			
I	None	0%	No residue
II	Trace	1–5%	Trace coating on the mucosa
III	Mild	5–25%	Epiglottic ligament visible
IV	Moderate	25–50%	Epiglottic ligament covered
V	Severe	> 50%	Filled to epiglottic rim
Pyriform sinus			
I	None	0%	No residue
II	Trace	1–5%	Trace coating on the mucosa
III	Mild	5–25%	Up wall to quarter full
IV	Moderate	25–50%	Up wall to half full
V	Severe	> 50%	Filled to aryepiglottic fold
